# Impact of the *ENPP1* mutation on bone mineralization and ectopic calcification: evidence from *in vitro* and *in vivo* models

**DOI:** 10.3389/fendo.2025.1566392

**Published:** 2025-06-04

**Authors:** Wanhong Wu, Luna Liu, Yingzhou Shi, Yidan Zhang, Renyuan Qiu, Fang Yan

**Affiliations:** ^1^ Key Laboratory of Endocrine Glucose & Lipids Metabolism and Brain Aging, Ministry of Education, Department of Endocrinology, Shandong Provincial Hospital Affiliated to Shandong First Medical University, Jinan, Shandong, China; ^2^ Department of Radiology, Shandong Rongjun General Hospital, Jinan, Shandong, China; ^3^ Department of Pain Management, Shandong Provincial Hospital Affiliated to Shandong First Medical University, Jinan, Shandong, China; ^4^ Department of Pain Management, Shandong Provincial Hospital, Cheeloo College of Medicine, Shandong University, Jinan, Shandong, China

**Keywords:** ectonucleotide pyrophosphatase/phosphodiesterase 1 (*ENPP1*), bone mineralization, ectopic calcification, diffuse idiopathic skeletal hyperostosis, ossification of the posterior longitudinal ligament

## Abstract

**Background:**

Ectonucleotide Pyrophosphatase/Phosphodiesterase 1 (*ENPP1*) plays a key role in mineralization processes, and mutations in this gene are associated with various severe diseases. Clinical case reports have implicated the *ENPP1* Y451C mutation in diffuse idiopathic skeletal hyperostosis patients, but its precise impact on bone mineralization and ectopic calcification remains unclear.

**Methods:**

We used bioinformatics tools and *in vitro* functional assays to assess the impact of the *ENPP1* Y451C mutation on protein structure and enzymatic activity. Furthermore, we generated a knock-in mouse model (*Enpp1^Y433C^*) to evaluate microarchitecture or signs of ectopic calcification by Micro-CT.

**Results:**

Bioinformatics analysis and *in vitro* assays showed that the Y451C mutation affects the *ENPP1* protein’s structure, reducing enzymatic activity by approximately 50%. We successfully generated the *Enpp1^Y433C^* knock-in mouse model. However, no significant differences were observed in body phenotype or biochemical markers in *Enpp1^Y433C^* mice at 3, 5, and 10 months, compared to wild-type controls. Similarly, no significant changes were observed in bone microarchitecture or signs of ectopic calcification.

**Conclusion:**

The *ENPP1* Y451C mutation significantly reduces enzymatic activity *in vitro*, yet the *Enpp1^Y433C^* knock-in mouse model shows no significant abnormalities in mineralization, providing additional evidence for the pathogenicity assessment of *ENPP1* Y451C variant. Given that these results are from mouse models, further studies are required to clarify its pathogenicity in humans.

## 1 Introduction

Ectonucleotide pyrophosphatase/phosphodiesterase (*ENPP1*) is a type 2 membrane-bound glycoprotein with a complex structure, featuring two key domains: extracellular catalytic and nuclease domain (1). It plays a crucial role in bone mineralization and soft tissue calcification, through hydrolyzing high-energy phosphate bonds to produce inorganic pyrophosphate (PPi), a potent inhibitor of ectopic mineral deposition (2, 3). Mutations in both alleles of the *ENPP1* are associated with generalized arterial calcification of infancy (GACI1: OMIM #208000) (4, 5) and autosomal recessive hypophosphatemic rickets type 2 (ARHR2: OMIM #613312) (6).

Studies have also implicated *ENPP1* deficiency in the development of ossification of the posterior longitudinal ligament (OPLL) and diffuse idiopathic skeletal hyperostosis (DISH). Both conditions are marked by excessive ossification in soft tissues, leading to pain, reduced mobility, and, in severe cases, spinal fractures or paralysis. The connection between *ENPP1* deficiency and OPLL was first proposed through studies on ttw (tip-toe walking) mice, which carry a loss-of-function mutation in the *ENPP1* gene and exhibit OPLL-like phenotypes (7, 8). Further evidence comes from findings in GACI survivors, where individuals with homozygous or compound heterozygous *ENPP1* mutations have been observed to develop ectopic bone formation at tendon and joint attachment sites at a relatively young age (9).

As reported in GACI and ARHR2, *ENPP1* deficiency is traditionally inherited in an autosomal recessive manner. However, emerging evidence suggests that *ENPP1* haploinsufficiency, even with a single functional allele, can also affect mineralization processes, contributing to early-onset osteoporosis, OPLL, and DISH (10, 11). Specifically, Hajime Kato et al. (10) identified a heterozygous *ENPP1* mutation (c.1352A>G, p.Y451C) in a 74-year-old female patient with DISH. This mutation, located in exon 13 within the catalytic domain, resides in a critical region essential for *ENPP1*’s enzymatic function. The patient’s CT imaging revealed ossification of the paraspinal ligaments, multiple spinal fractures, and mild ectopic calcification in the Achilles tendon. Biochemical analysis showed low-normal serum phosphate levels and elevated fibroblast growth factor 23 (FGF23) levels, which are characteristic of *ENPP1* deficiency. Notably, Hajime Kato et al. (10) also reported that the same heterozygous *ENPP1* p.Y451C mutation was identified in two younger brothers (aged 19 and 23), both of whom developed calcific Achilles tendon enthesopathies. Further enzymatic assays revealed a 70% reduction in *ENPP1* activity due to the Y451C mutation, linking the biochemical profile and reduced enzyme activity to the disease phenotypes. We also observed a compound *ENPP1* mutation (c.783C>G/c.1352A>G, p.Y243X/p.Y451C) in a boy who presented with low-normal serum phosphate levels and hoof-like feet and was ultimately diagnosed with ARHR2. However, his mother, a carrier of the Y451C mutation, currently exhibits no clinical symptoms, highlighting the variability in phenotypic expression.

Building on these findings, we aimed to further explore the contribution of *ENPP1*, especially the Y451C mutation, to the development of OPLL and DISH. Using bioinformatics tools and *in vitro* functional assays, we assessed the mutation’s impact on *ENPP1*’s structure and enzymatic activity. Moreover, we generated a knock-in mouse model (*Enpp1^Y433C^*) to investigate the *in vivo* effects of this mutation, with particular focus on its impact on bone and soft tissue mineralization. This study aims to clarify the pathogenicity of *ENPP1* Y451C mutation, providing insights for genetic counseling and risk assessment.

## 2 Materials and methods

### 2.1 Bioinformatic analysis

*ENPP1* protein structures (6WFJ) were obtained from the PDB (https://www.rcsb.org/). To evaluate the potential impact of the mutation on protein structure, predictions of the mutant’s tertiary structure were predicted using PyMOL (version 1.3). Multiple sequence alignment was measured by using Clustal Omega (https://www.ebi.ac.uk/Tools/msa/clustalo/).

### 2.2 Cell culture and transfection

Human embryonic kidney 293T cells and murine preosteoblastic MC3T3-E1 cells were cultured in Dulbecco’s Modified Eagle Medium (DMEM) and alpha-Minimum Essential Medium (α-MEM), respectively. The cells were maintained at 37°C in a humidified atmosphere with 5% CO2. Upon reaching 80-90% confluence, the cells were passaged using 0.05% trypsin-EDTA.The full length of the major transcript of human *ENPP1* (transcript ID: NM_006208) was synthesized and cloned into the transient overexpression vector GV141 (GeneChem, China). The *ENPP1* Y451C mutant plasmid was introduced into the plasmid construct using a site-directed mutagenesis approach. Constructs encoding *ENPP1*(WT) and *ENPP1*-Y451C were tagged with a FLAG epitope for detection. For transfection, cells at 70-80% confluence were transfected with plasmids using Lipofectamine 3000, following the manufacturer’s instructions. After transfection for 48 hours, the cells were harvested for further analysis, including protein expression and functional assays.

### 2.3 *Enpp1^Y433C^* Cas9-KI mice generation

All the animal experimental protocols were approved by the Institutional Animal Care and Use Committee at Shandong Provincial Hospital in accordance with institutional guidelines for the use of laboratory animals (approval number LCYJ: No. 2019-147). The A-to-G substitution in the *Enpp1* gene was introduced using CRISPR/Cas9-mediated knock-in in mice with a C57BL/6J background. Two additional base changes were introduced to create a BsaI restriction site for genotyping. Sequences near the Y433 codon in exon 13 of the *Enpp1* gene (ID: 18605) were screened and transcribed into sgRNAs. The sgRNA efficiency was evaluated by Cas9 microinjection into zygotes and subsequent blastocyst-stage culture. The resulting pups were genotyped by sequencing a PCR amplicon generated using the following primers: 5’TCTGGAGGTATCAGGTGGCTGA3’ (forward) and 5’AGATTACCACATAGTGTCCGGTCC3’ (reverse). Mice were maintained on a 12-hour dark/light cycle, on regular chow, and food and water ad libitum. Phenotypic analyses were performed on male mice at 3, 5, and 10 months of age, with each group consisting of 5–6 samples.

Blood samples were collected by eyeball extirpation under anaesthetization, then centrifuged to collect serum and stored at −80°C for further biochemical analysis. Left femurs were fixed in paraformaldehyde and used for micro-computed tomography (CT) analysis. Right femurs were wrapped in saline-soaked gauze for a 3-point bending mechanical test.

### 2.4 Enzyme kinetic assays

*ENPP1* enzyme kinetic assays were performed as previously described (10). After transfection for 48 hours, 10 µL of cell culture supernatant was mixed with 90 µL of assay buffer containing 250 mM Tris (pH 8.0), 500 mM NaCl, 0.05% Triton X-100, and 1 mM thymidine 5′-monophosphate (TMP). The reaction was initiated, and the release of p-nitrophenol from the substrate was measured at an optical density (OD) of 405 nm. The results were normalized to a percentage of the wild-type (WT) enzyme activity.

### 2.5 Biochemistry assays

Blood serum was prepared and assayed for Pi, Ca, Mg and ALP levels using an automatic biochemical analyzer (BS-830, Mindray, China). Intact FGF23 was measured using mouse FGF23 Elisa kits (SenBeiJia Biological Technology Co., Ltd., RRID: AB_3076185), in accordance with the manufacturer’s protocol.

### 2.6 X-ray

Whole-body X-rays of mice were captured using a DR uDR588i (United Imaging Corp., China) under standardized settings (50 kV, 40 ms). Images were analyzed from DICOM files using RadiAnt DICOM Viewer software (Poznan, Poland).

### 2.7 Micro-computed tomography

For bone microstructure evaluation, the left femurs were dissected to remove soft tissue and fixed in 4% paraformaldehyde, and then scanned according to the reported guideline (12). Scanning was conducted at 70 kVp and 114 μA, with a 250 ms integration time and a 15.6 μm isotropic voxel size (vivaCT60, Scanco Medical). Trabecular bone was analyzed in 100 slices from the distal end of the femur to the distal growth plate. Primary trabecular parameters included trabecular bone volume/total volume (Tb.BV/TV), trabecular number (Tb.N), trabecular thickness (Tb.Th), trabecular spacing (Tb.Sp). Cortical bone was in the middle 100 slices of the femoral diaphysis. Primary cortical parameters included cortical bone volume/total volume (Cb.BV/TV), cortical thickness (Ct.Th), and cortical density (Ct. BMD) using a μCT-80 scanner (vivaCT60, Scanco Medical). Additionally, whole-body and hind paw scans of the mice were performed.

### 2.8 Biomechanical test

3-point bending was used to determine biomechanical characteristics. The Right femurs were thawed, kept moist in saline, and loaded to fracture in the anterior-posterior direction. A load rate of 0.1 mm/s and a preload of 0.5 N were applied to each bone to prevent shifting during testing using a servo hydraulic testing machine (3230 SERIES III, WATERS Corp). Load-displacement curves were used to calculate the maximum load and bending stiffness.

### 2.9 Western blotting

Western blotting was used to evaluate *ENPP1* protein expression in HEK293 cells transfected with wild-type or mutant *ENPP1* constructs, as well as in mouse bone tissue. Total protein was extracted from cells and tibial tissue using RIPA buffer. Proteins were separated by SDS-PAGE, transferred to a PVDF membrane, and blocked with 5% non-fat milk. The membrane was incubated with primary antibodies against FLAG (1:1000, # 14793S, Cell Signaling Technology), ACTIN (1:7500, #66009-1-Ig, Proteintech) and *ENPP1* (1:1000, #2061S, Cell Signaling Technology), followed by HRP-conjugated secondary antibodies. Protein bands were visualized using an ECL system to assess *ENPP1* expression levels.

### 2.10 Immunofluorescence and confocal microscopy

Following transfection, cells were fixed with 4% paraformaldehyde for 15 minutes, permeabilized with 0.1% Triton X-100 in PBS for 10 minutes, and blocked with 5% BSA in PBS for 1 hour to reduce non-specific binding. FLAG and ATP1A1 epitopes were detected using anti-FLAG (1:500, Sigma-Aldrich) and anti-ATP1A1 (1:500, Proteintech) antibodies, incubated overnight at 4°C. After three washes with PBS, cells were incubated with fluorescent secondary antibodies (Alexa Fluor 488, 555, 1:1000) for 1 hour. After further washes with PBS, the samples were mounted with a mounting medium containing DAPI Confocal images were acquired using a confocal laser scanning microscope (Leica TCS SP8) to assess the localization of the FLAG-tagged *ENPP1* constructs.

### 2.11 Statistical analysis

Values are expressed as mean ± standard deviation (SD), as indicated in figure legends. Statistical analysis was performed using GraphPad Prism 9, with T-tests for two-group comparisons and one-way ANOVA for comparisons among three groups. P values < 0.05 were considered to statistically significant.

## 3 Result

### 3.1 Bioinformatics analysis of *ENPP1* Y451C mutation

**Figure 1:**
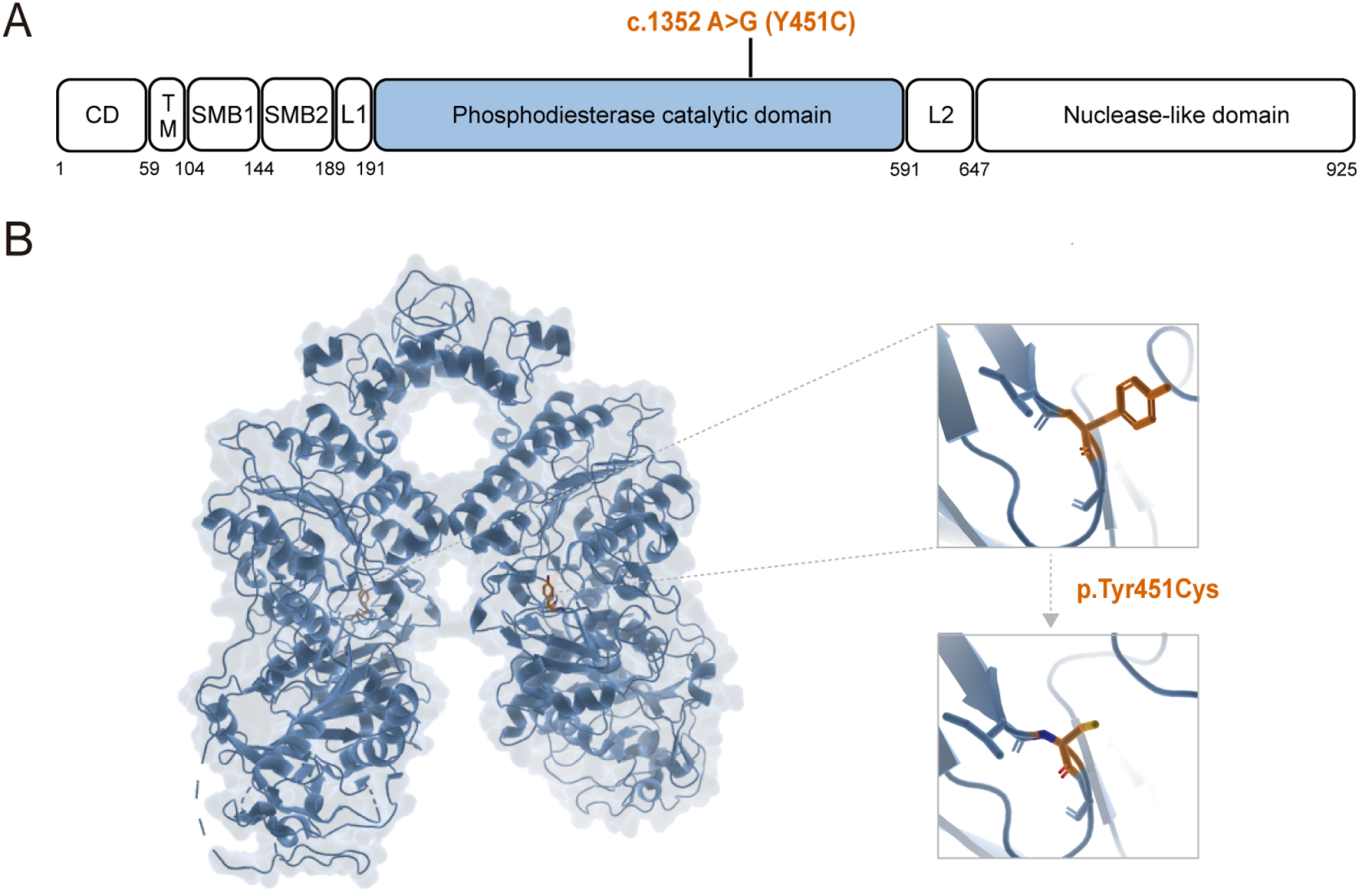
Structure prediction of *ENPP1* Y451C mutation. **(A, B)** *ENPP1* structure and the location of the Y451C variants.

The p.Y451C mutation occurs within the catalytic domain of the *ENPP1* protein (**Figure 1A**), which is crucial for its enzymatic function. To assess the potential structural impacts of this mutation, we utilized the PyMOL molecular visualization program to predict alterations in the protein’s three-dimensional conformation (**Figure 1B**). The Y451C variant disrupts the alpha-helix structure, which may impair the protein’s functionality by affecting its modification processes or its interactions with other proteins and ions (**Figure 1B**). Additionally, a sequence alignment analysis across five species revealed that the Y451 position is highly conserved, indicating its evolutionary importance and suggesting that this site is functionally significant across different organisms (**Supplementary Figure S1**).

### 3.2 Functional characterization of *ENPP1* Y451C mutation *in vitro*

**Figure 2:**
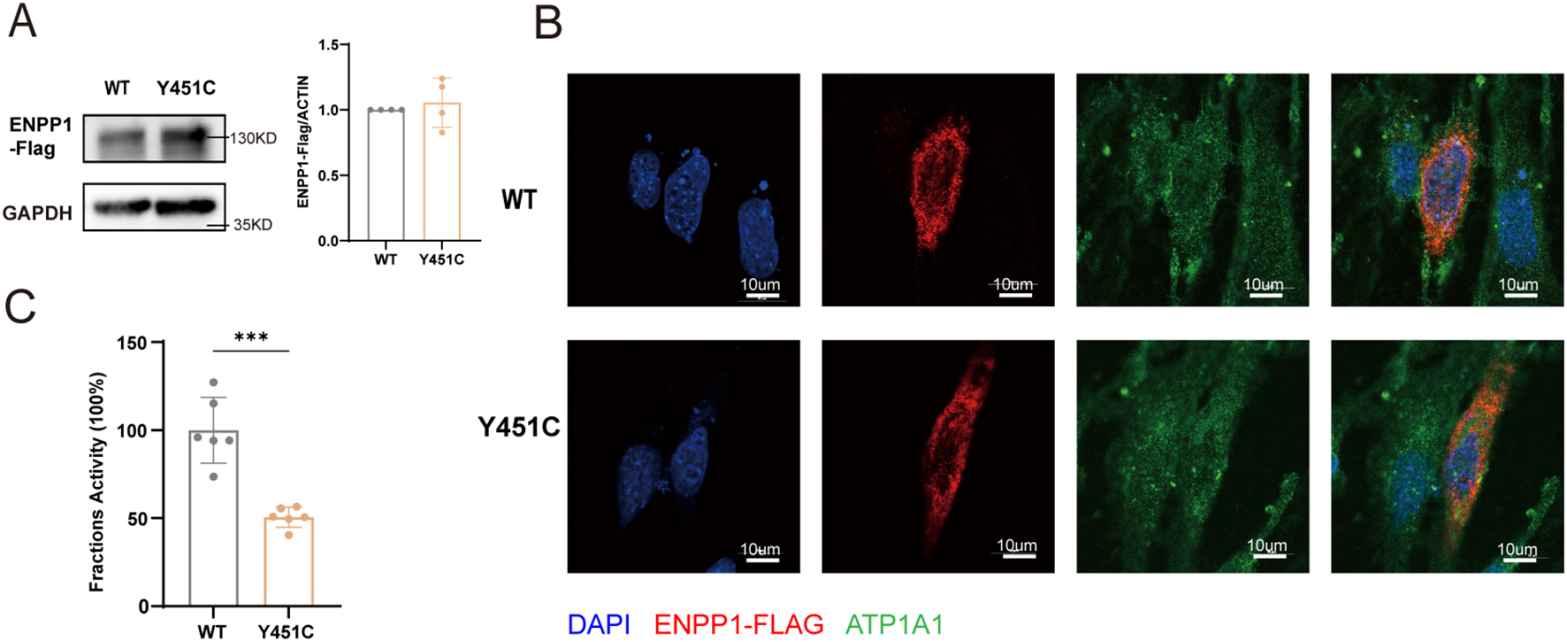
Functional analysis of *ENPP1* Y451C mutation *in vitro*. **(A)** Western blotting and quantification of *ENPP1* protein expression in HEK293T cells transfected with *ENPP1* wild-type (WT) and Y451C plasmids. **(B)** Immunofluorescence imaging showing the expression and localization of *ENPP1* in MC3T3-E1 cells. **(C)** Enzymatic activity in HEK293T cells. Data are expressed as mean ± standard deviation (n=4-6). Statistical analysis was performed using unpaired student's t-tests for comparisons between two groups, *** p<0.001.

To further investigate the pathogenic role of *ENPP1* Y451C mutation on protein function, we transfected HEK293T cells with an overexpression plasmid containing the wild-type (WT) and mutation. We used Western blotting to determine whether the mutation affected *ENPP1* protein expression levels. The results indicated that the mutation did not alter protein expression compared to WT (**Figure 2A**).

Given that *ENPP1* functions as a transmembrane protein, we utilized immunofluorescence microscopy to examine the localization of the *ENPP1* protein in osteoblast-like MC3T3-E1 cells. Our observations indicated that the Y451C mutation did not alter protein localization in the cellular (**Figure 2B**). We then proceeded to evaluate the enzymatic activity of the *ENPP1* protein. Consistent with findings reported by Hajime Kato et al. (10), the Y451C mutation led to approximately a 50% reduction in enzymatic activity compared to WT (**Figure 2C**), suggesting that the decrease in enzymatic activity is not due to alterations in protein expression or localization.

### 3.3 Design of the *Enpp1^Y433C^* mouse model

**Figure 3:**
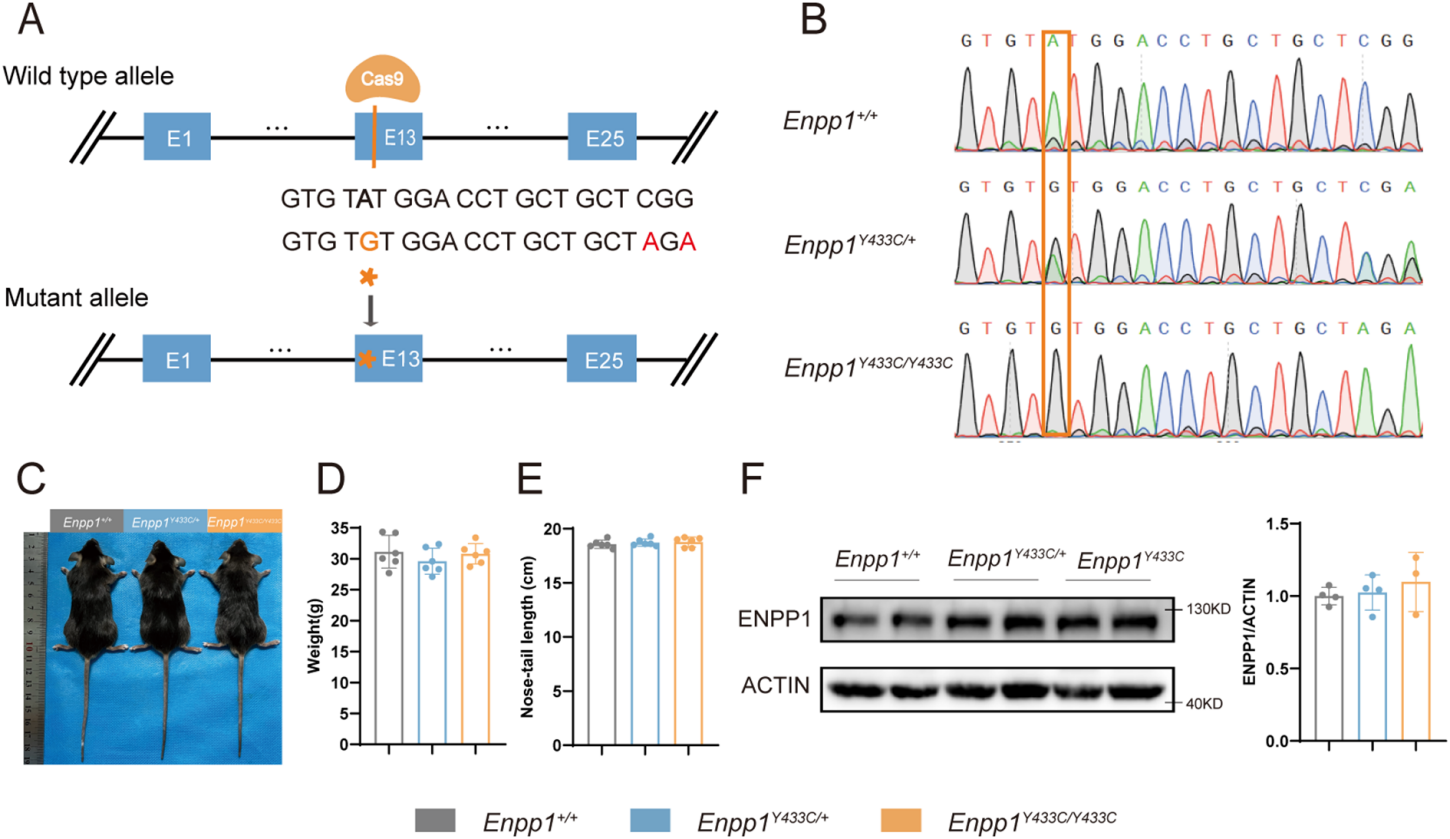
Generation of the *Enpp1^Y433C^* mutant mouse. **(A)** Design methods of *Enpp1^Y433C^* knock-in mice were performed by CRISPR-Cas9. **(B)** Genotyping of *Enpp1^Y433C^* via sequencing, the red box highlights the mutation site (A>G). **(C-E)** Whole-body photographs, body weight and nose-tail length measurements of 10-month-old male mice. **(F)** Western blotting and quantification of *ENPP1* protein expression in tibial tissue from 10-month-old male mice. *ENPP1* protein levels were normalized to ACTIN. Data are expressed as mean ± standard deviation(n=3-6). Statistical analysis was performed using one-way ANOVA for comparisons among three groups.

We engineered an *Enpp1* knock-in mouse model using CRISPR-Cas9 gene-editing technology to introduce a specific point mutation (A>G) (**Figure 3A**), resulting in the substitution of amino acid tyrosine with cysteine at position 433, mimicking the mutation observed in human patients (Y451C). Genetically modified embryos were implanted into pseudo-pregnant C57BL/6 females, leading to the birth of pups that were genotyped to identify those heterozygous for the Y433C mutation. These heterozygous mice were then backcrossed with C57BL/6 mice to produce the F2 generation, resulting in wild-type (WT) and homozygous mutant (*Enpp1^Y433C^*) sibling pairs, validated through PCR and Sanger sequencing (**Figure 3B**). No significant differences were observed in body weight or length between the groups (**Figures 3C–E**). Furthermore, comparative analysis of *ENPP1* protein levels in tibial samples via western blotting showed no significant differences in expression when compared to WT (**Figure 3F**), consistent with cellular results, which demonstrate that specific Y433C mutations did not affect overall protein expression levels.

### 3.4 Phenotypic manifestations and serum analytes of *Enpp1^Y433C^* mice

**Figure 4:**
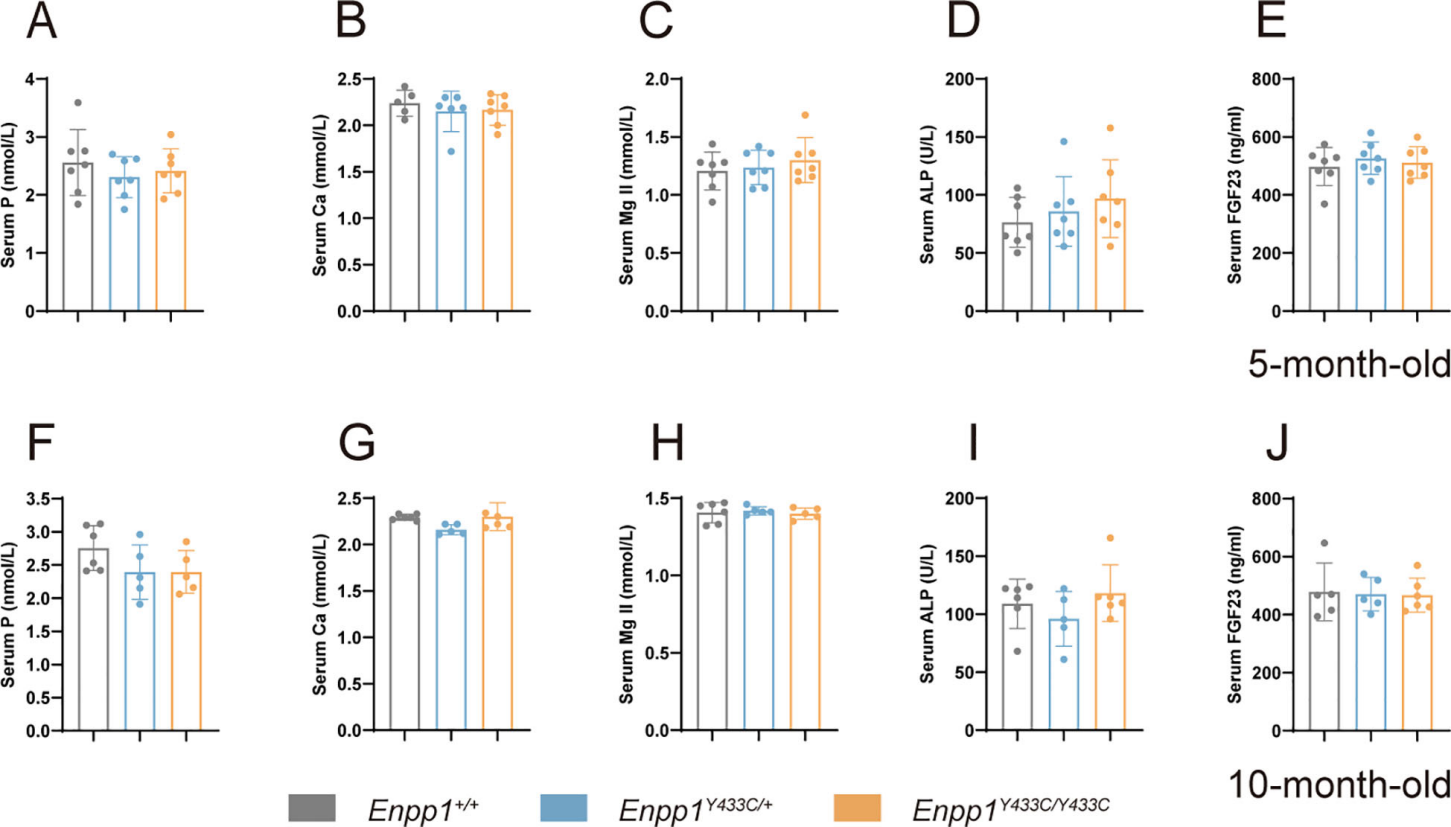
Biochemistry analysis in 5- and 10- months-old male mice. **(A-E)** Biochemistry features of phosphate (P), calcium (Ca), magnesium (Mg), alkaline phosphatase (ALP) and FGF23 levels in 5- month-old mice. **(F-J)** Biochemistry features of phosphate (P), calcium (Ca), magnesium (Mg), alkaline phosphatase (ALP) and FGF23 levels in 10-month-old mice. Data are expressed as mean ± standard deviation(n=5-7). Statistical analysis was performed using one-way ANOVA for comparisons among three groups.

Low-normal serum phosphate and high-normal FGF23 levels were observed in the female patient with the heterozygous Y451C mutation, consistent with the known effects of *ENPP1* deficiency (10). Initially, we analyzed the biochemical features of 3-month-old *Enpp1^Y433C^* mice, but no significant differences were observed (**Supplementary Figures S2A–E**). Given that *ENPP1* mutations are known to lead to progressively worsening phenotypes, we extended our analysis to 5-month-old mice. Serum analytes in *Enpp1^Y433C^* mice showed no significant alterations in key factors related to bone mineralization, such as phosphate (P), calcium (Ca), and alkaline phosphatase (ALP), compared to WT mice (**Figures 4A–D**). Similarly, serum levels of FGF23, a critical regulator of phosphate metabolism, remained unchanged in *Enpp1^Y433C^* mice (**Figure 4E**). Analysis of 10-month-old mice revealed similar results, with no significant changes in these parameters (**Figures 4F–J**).

**Figure 5:**
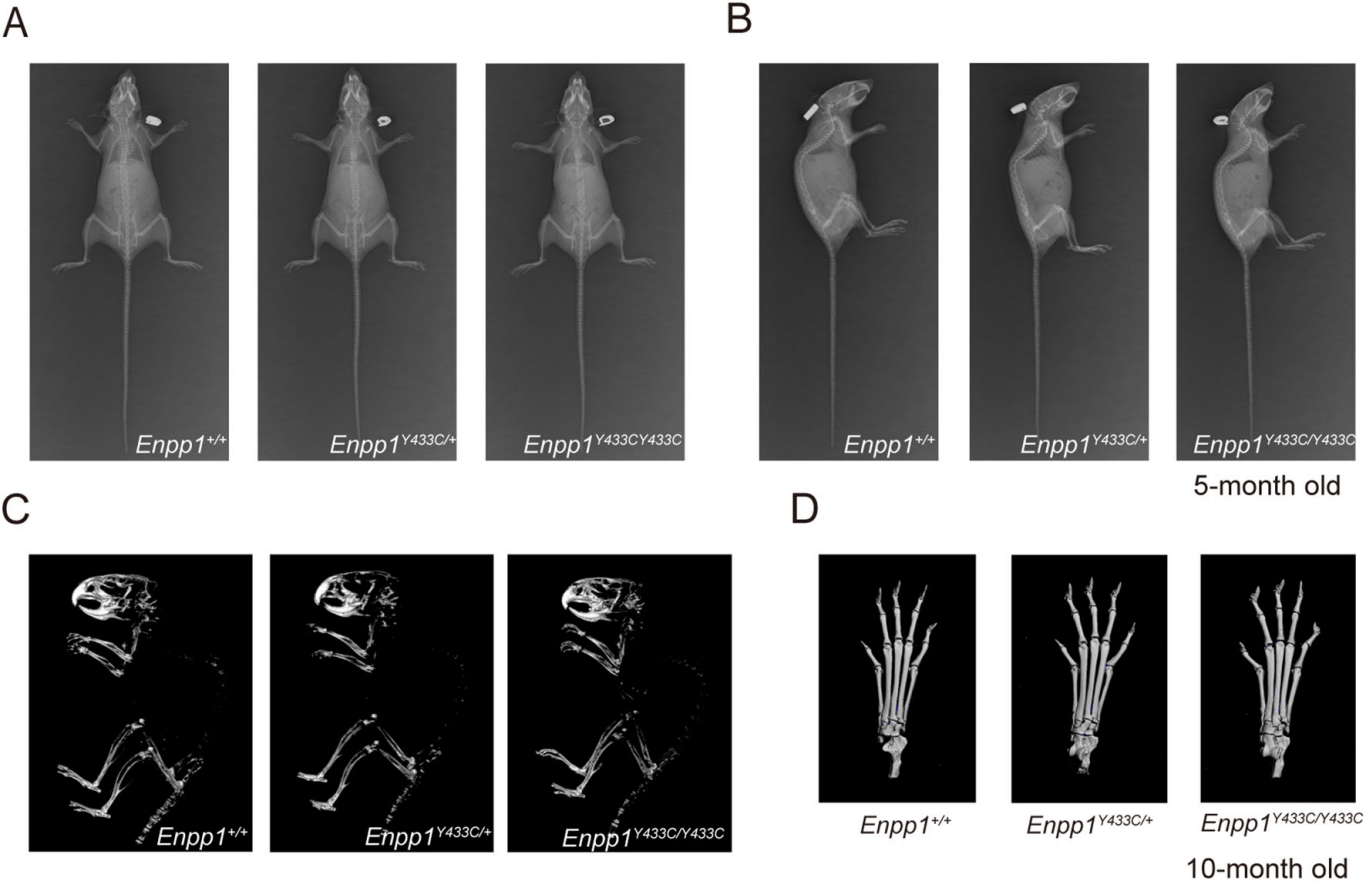
Ectopic calcification analysis in 5- and 10- months old male mice. **(A, B)** X-ray images of *Enpp1^Y433C^* mice in both anterior-posterior and lateral views. **(C, D)** 3D reconstruction of whole-body and paw imaging.

Ossification of the paraspinal ligaments and mild ectopic calcification in the Achilles tendon were observed in the female patient with the Y451C heterozygous mutation on CT imaging. We first assessed calcification in 5-month-old *Enpp1^Y433C/+^* and *Enpp1^Y433C^* mice using X-rays, but no ectopic calcification in the spine or paws was detected compared to the WT group (**Figures 5A, B**). Further micro-CT analysis of 10-month-old *Enpp1^Y433C^* mice still revealed no ectopic calcifications around the joints in the forepaws (**Figures 5C, D**).

### 3.5 Bone microarchitecture of *Enpp1^Y433C^* mice

**Figure 6:**
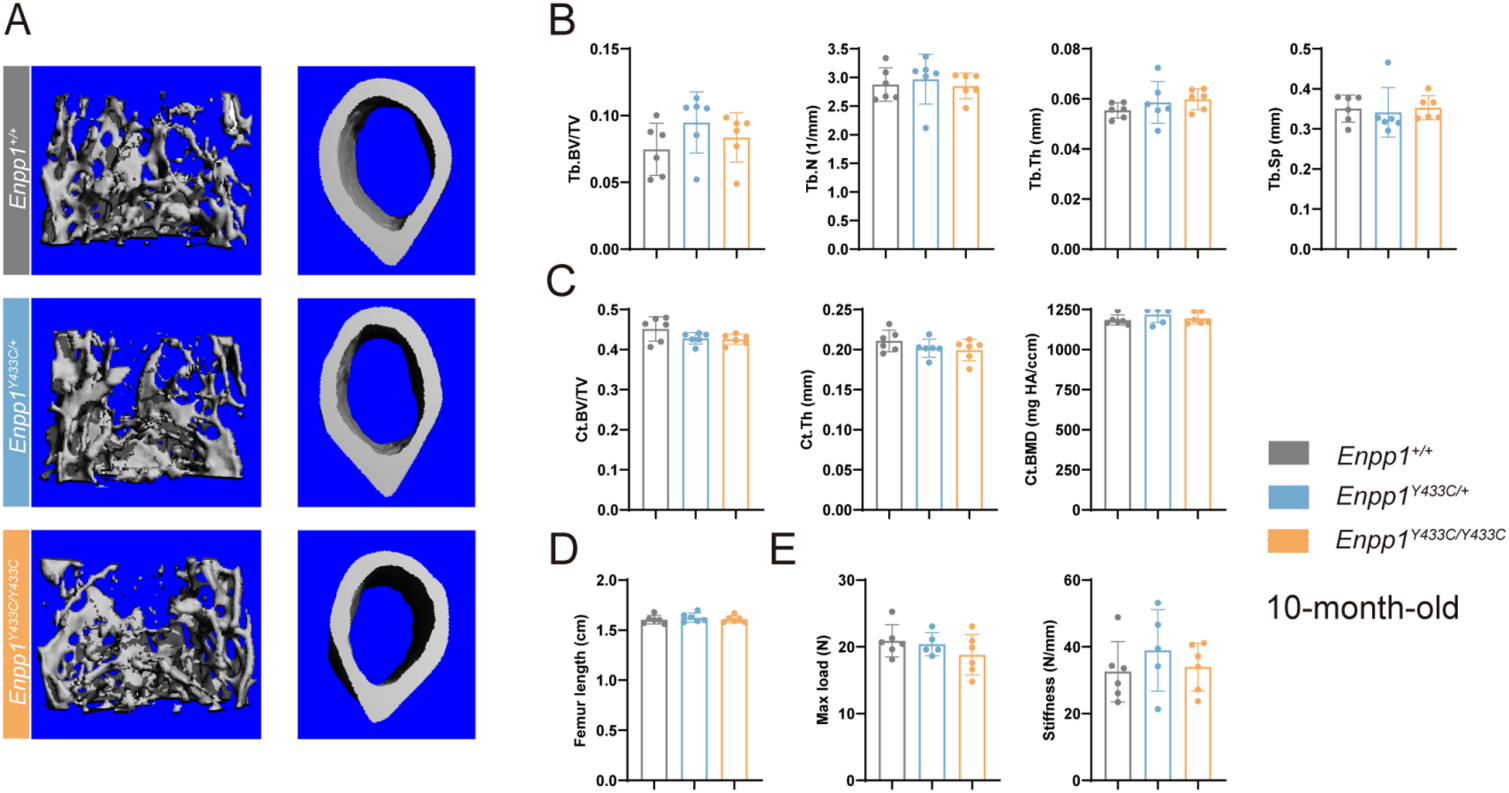
Bone microarchitecture and biomechanics of 10-month-old male mice. **(A)** Three-dimensional reconstructed images of femoral trabecular and cortical bone. **(B)** Quantification of trabecular BV/TV, trabecular number (Tb.N), trabecular thickness (Tb.Th), trabecular spacing (Tb.Sp). **(C)** Quantification of cortical BV/TV, cortical thickness (Ct.Th), and cortical density (Ct. BMD). **(D)** Femur length. **(E)** Quantification of femur biomechanical properties (maximum load, stiffness). Data are expressed as mean ± standard deviation(n=5-6). Statistical analysis was performed using one-way ANOVA for comparisons among three groups.

To assess bone mineralization, we analyzed femoral bone microarchitecture using micro-CT in 3-month-old mice. No significant differences in trabecular and cortical bone structure were observed in *Enpp1^Y433C^* and *Enpp1^Y433C/+^* mice compared to the WT group (**Supplementary Figures S3A–D**), despite the presence of vertebral compression fractures in a female patient with the Y451C heterozygous mutation, indicative of osteoporosis (10). We then extended our analysis to 5-month-old (**Supplementary Figures S4A–F**) and 10-month-old mice, microarchitectural analysis revealed no significant bone mass loss in *Enpp1^Y433C^* and *Enpp1^Y433C/+^* mice. Compared to their WT sibling pairs, there were no significant differences in trabecular BV/TV, trabecular number (Tb.N), thickness (Tb.Th) and spacing (Tb.Sp). Similarly, cortical BV/TV, thickness (Ct.Th) and density (Ct. BMD) remained unaffected (**Figures 6A–C**). Additionally, femur length did not differ significantly among heterozygous, homozygous, and WT groups (**Figure 6D**). Furthermore, femur maximum load or stiffness also exhibited no notable differences (**Figure 6E**). Similarly, no significant differences were observed in the 10-month-old female mice, compared to the WT group (**Supplementary Figure S5** **).**

These findings show that the *Enpp1^Y433C^* mutation does not reduce bone mass in mice, consistent with studies showing the *Enpp1^T238A^* mutation does not affect bone microstructure (13), highlighting the non-catalytic role of *ENPP1* in the regulation of skeletal health.

## 4 Discussion

To validate the pathogenicity of the mutation, we generated an *Enpp1^Y433C^* knock-in mouse model. Regrettably, our findings did not align with those observed in the DISH patients. Although the *ENPP1* Y451C mutant patient has revealed ectopic calcification and multiple spinal fractures, our data show that *Enpp1^Y433C^* mutation mice do not exhibit significant changes in ectopic calcification and bone mineralization.

Mutations in the *ENPP1* gene are associated with various diseases. As a key regulator of PPi, *ENPP1* encodes an enzyme that hydrolyzes ATP to produce PPi, playing a crucial role in the regulation of mineralization (14). GACI represents the most severe form of *ENPP1* deficiency (4). Subsequent studies have identified phenotypes such as ARHR2, DISH, and OPLL in survivors of GACI (9). Recent case reports have also linked *ENPP1* heterozygous and compound heterozygous mutations to early-onset osteoporosis, DISH, and OPLL (10, 15, 16). Additionally, *ENPP1* has also been linked to insulin resistance, type 2 diabetes (17), Cole disease (18), cancer metastasis, and osteoarthritis (19), highlighting its functional significance and the heterogeneity of *ENPP1* mutation-related phenotypes.

*Enpp1^ttw^*, a classic mouse model of ossification of the posterior longitudinal ligament of the spine, was demonstrated by (8). *Enpp1^ttw^* has a c.1702G>T substitution, resulting in a nonsense mutation (p.G568X) in the *Enpp1* coding region, which exhibits posterior longitudinal ligament ossification and a tip-toe walking phenotype, accompanied by widespread soft tissue calcification and reduced blood Pi levels (8, 20). The point mutation mouse model *Enpp1^Y433C^* we developed, was derived from clinical *ENPP1* Y451C mutation patients, but it does not exhibit the phenotypes observed in clinical patients. There are no marked differences in serum analytes such as Pi, ALP, and FGF23 in *Enpp1^Y433C/+^* and *Enpp1^Y433C^* mice. Furthermore, no ectopic calcification was observed, and there were no significant changes in bone microarchitecture, even at advanced ages. However, our *in vitro* results, showing that the *ENPP1* Y451C variant leads to a 50% reduction in enzyme activity, align with those of Hajime Kato et al (10). In contrast, the *Enpp1^T238A^* mutation, which completely abolishes *ENPP1* enzyme activity (21), results in low plasma Pi and PPi levels, elevated FGF23, and significant ectopic calcification (13). *Enpp1^T238A^* mice exhibit normal trabecular microarchitecture, but cortical BV/TV and cortical thickness are reduced at 23 weeks (13). Considering these differences, this suggests the possibility of compensatory mechanisms in mice that mitigate the effects of reduced *ENPP1* enzyme activity.

*ENPP1* is one of the members of the ENPP family, and the physiological functions of its members1–7 are primarily determined by their substrate specificity and tissue-specific expression levels (14, 22). Among them, *ENPP1* and ENPP3 share structural and functional similarities, both hydrolyzing ATP to produce AMP and inorganic pyrophosphate (PPi) (14, 23). *ENPP1* is widely expressed throughout the body, while ENPP3 is predominantly expressed in immune cells and tumor tissues. ​Yano Y et al. also reported that ENPP3 can be expressed in hepatocytes (24–26); we may infer that ENPP3 in mouse liver cells hydrolyzes ATP to generate PPi. Additionally, the main regulators of PPi include *ENPP1*, ATP-binding cassette sub-family C member 6 (ABCC6), tissue-nonspecific alkaline phosphatase (TNAP), and the PPi channel protein ANK. As an ATP transporter on the basal membrane of hepatocytes, ABCC6 regulates PPi levels by facilitating the release of ATP into the bloodstream (27). *ENPP1* hydrolyzes ATP to generate PPi, which in turn inhibits the formation and deposition of HA crystals, while TNAP hydrolyzes PPi into Pi to promote HA formation (28). ANK is a transmembrane protein that directly transports intracellular PPi to the extracellular matrix (29). These four proteins together form a mineralization regulatory network that provides the correct PPi/Pi ratio for physiological mineralization. These factors may potentially alleviate the reduction in PPi production caused by *ENPP1* deficiency by increasing PPi levels.

Furthermore, the pathogenesis of DISH and OPLL is complex, involving multiple genetic factors and gene-gene interactions. GWAS has identified multiple susceptibility genes linked to DISH and OPLL. Variants in COL6A1 (30), BMP2 (31, 32), and TGFβ1 (33) are associated with both the occurrence and severity of OPLL. Notably, certain SNPs in BMP2 and TGFβ1 (33) also appear in DISH patients, suggesting their involvement in DISH pathogenesis as well. Additionally, FGF23-related hypophosphatemia has been closely associated with the development of spinal ligament ossification. X-linked hypophosphatemia (XLH), caused by mutations in the PHEX gene, disrupts FGF23-mediated phosphate regulation, thereby increasing the risk of OPLL. Studies indicate that approximately 71% of XLH patients develop OPLL (34). Similarly, Autosomal Recessive Hypophosphatemic Rickets Type 1 is also associated with spinal ligament ossification (35).

Non-genetic factors may also act as triggers or modifiers, influencing the progression of DISH and OPLL. While genetic factors play a dominant role in the pathogenesis of these disorders, environmental factors, particularly obesity and metabolic diseases, may exacerbate the risk of spinal ligament ossification. Recent studies suggest that chronic low-grade inflammation caused by obesity may promote the calcification of spinal ligaments, while elevated levels of IGF-1 may accelerate the ossification process by enhancing bone formation and tissue proliferation (36, 37). Furthermore, the incidence of OPLL varies significantly across different populations, with a notably higher prevalence in East Asian populations. In Japan, the prevalence of OPLL ranges from 1.9% to 4.3%, while it remains much lower in Western populations (0.01%–2.0%) (38, 39). This disparity may be partially attributed to differences in environmental factors or lifestyle.

Our findings reveal marked discrepancies between the *Enpp1^Y433C^* murine model and clinical manifestations in human patients. While *ENPP1 Y451C* reduced enzymatic activity *in vitro*, the *Enpp1^Y433C^* mutation mice exhibit a limited effect on ectopic calcification and bone mineralization. This suggests the potential existence of compensatory mechanisms in mice and also highlights the complexity of the pathogenesis of DISH and OPLL, which involves a complex interplay of genetic and environmental factors. According to the ACMG guidelines (40), our *in vitro* data (aligned with Hajime Kato et al.’s findings (10)), provide strong pathogenic (PS3) evidence for the *ENPP1* Y451C variant, while the *Enpp1^Y433C^* murine model suggests strong benign (BS3) evidence, presenting a contradiction. Our findings provide additional evidence for the pathogenicity assessment of *ENPP1* Y451C variant. There are some limitations in our study. Although the amino acid at the mutation site is conserved across species, species-specific genetic and physiological differences between mice and humans may impact phenotypic outcomes. Additionally, the controlled and uniform housing conditions of mice cannot fully replicate the complex environmental factors that contribute to disease manifestation in humans. Future studies should aim to collect more cases with this mutation to provide stronger evidence clarifying its pathogenicity in humans, thereby facilitating genetic counseling and helping patients and their families better understand the associated genetic risks.

## 5 Conclusion

The *ENPP1* Y451C mutation significantly reduces enzymatic activity *in vitro*; however, the *Enpp1^Y433C^* mutation in mice exhibits a limited effect on ectopic calcification and bone mineralization. These findings provide additional evidence for the pathogenicity assessment of *ENPP1* Y451C variant. Given that these results are derived from mouse models, which may not fully recapitulate human disease, future studies should focus on collecting more cases with this mutation to clarify its pathogenicity in humans and improve genetic counseling.

## Data Availability

The original contributions presented in the study are included in the article/**Supplementary material**. Further inquiries can be directed to the authors.
