# Supplementary material for: Impact of the ENPP1 mutation on bone mineralization and ectopic calcification: evidence from in vitro and in vivo models
Source: Front Endocrinol (Lausanne). 2025 Jun 4;16:1566392. doi: 10.3389/fendo.2025.1566392 (PMC12173856; doi:10.3389/fendo.2025.1566392)
Supplement: Supplementary file 1 [file DataSheet1.docx]

**Supplementary Material**


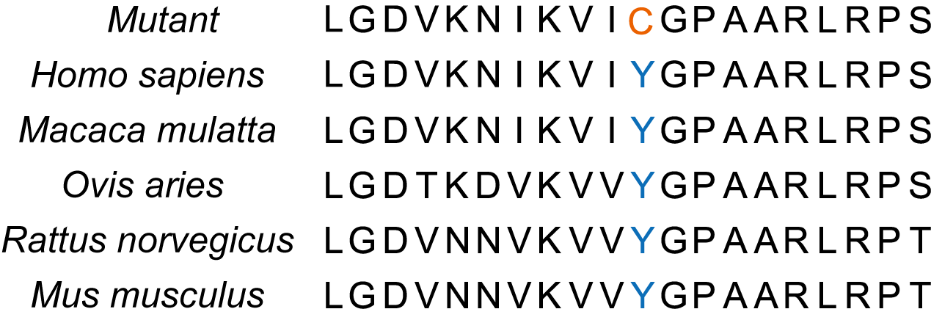


**Figure S1. Conservation of the Y451 position across five species.**


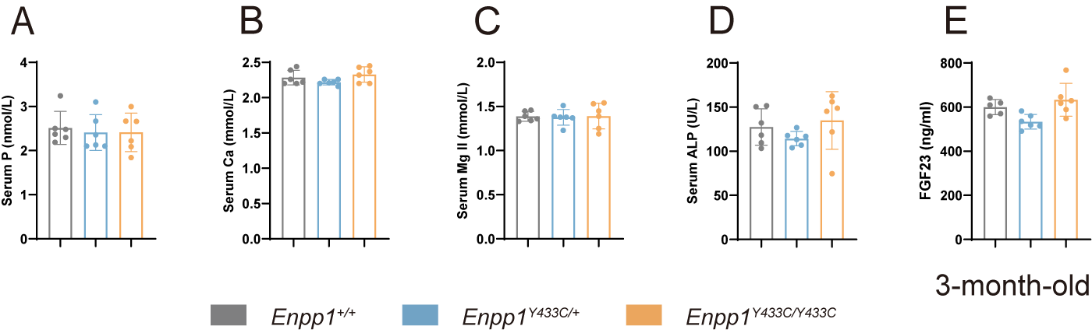


**Figure S2. Biochemistry analysis in 3-month-old male mice.**

(A-E) Biochemistry features of phosphate (P), calcium (Ca), magnesium (Mg), alkaline phosphatase (ALP) and FGF23 levels in 3- month-old mice. Data are expressed as mean ± standard deviation(n=6). Statistical analysis was performed using one-way ANOVA for comparisons among three groups.


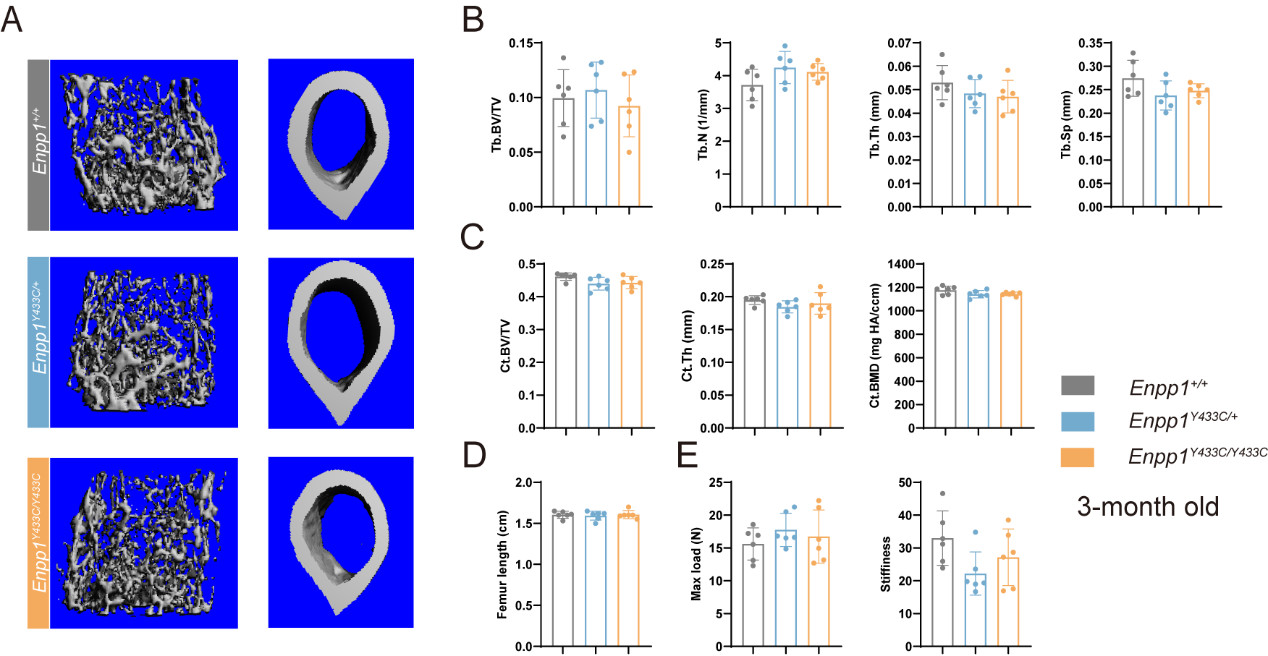


**Figure S3. Bone microarchitecture and biomechanics of 3-month-old male mice.**

(A) Three-dimensional reconstructed images of femoral trabecular and cortical bone. (B) Quantification of trabecular BV/TV, trabecular number (Tb.N), trabecular thickness (Tb.Th), Trabecular spacing (Tb.Sp). (C) Quantification of cortical BV/TV, cortical thickness (Ct.Th), and cortical density (Ct. BMD). (D) Femur length. (E) Quantification of femur biomechanical properties (maximum load, stiffness). Data are expressed as mean ± standard deviation(n=6). Statistical analysis was performed using one-way ANOVA for comparisons among three groups.


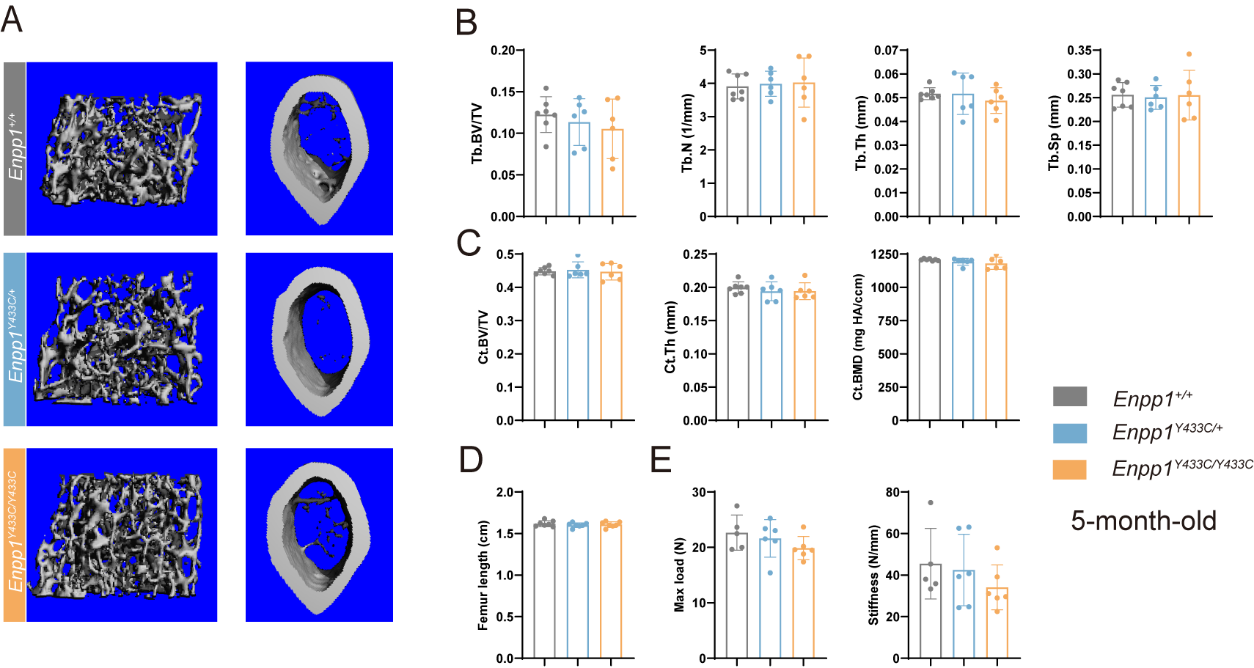


**Figure S4. Bone microarchitecture and biomechanics of 5-month-old male mice.**

(A) Three-dimensional reconstructed images of femoral trabecular and cortical bone. (B) Quantification of trabecular BV/TV, trabecular number (Tb.N), trabecular thickness (Tb.Th), Trabecular spacing (Tb.Sp). (C) Quantification of cortical BV/TV, cortical thickness (Ct.Th), and cortical density (Ct. BMD). (D) Femur length. (E) Quantification of femur biomechanical properties (maximum load, stiffness). Data are expressed as mean ± standard deviation(n=5-6). Statistical analysis was performed using one-way ANOVA for comparisons among three groups.

**
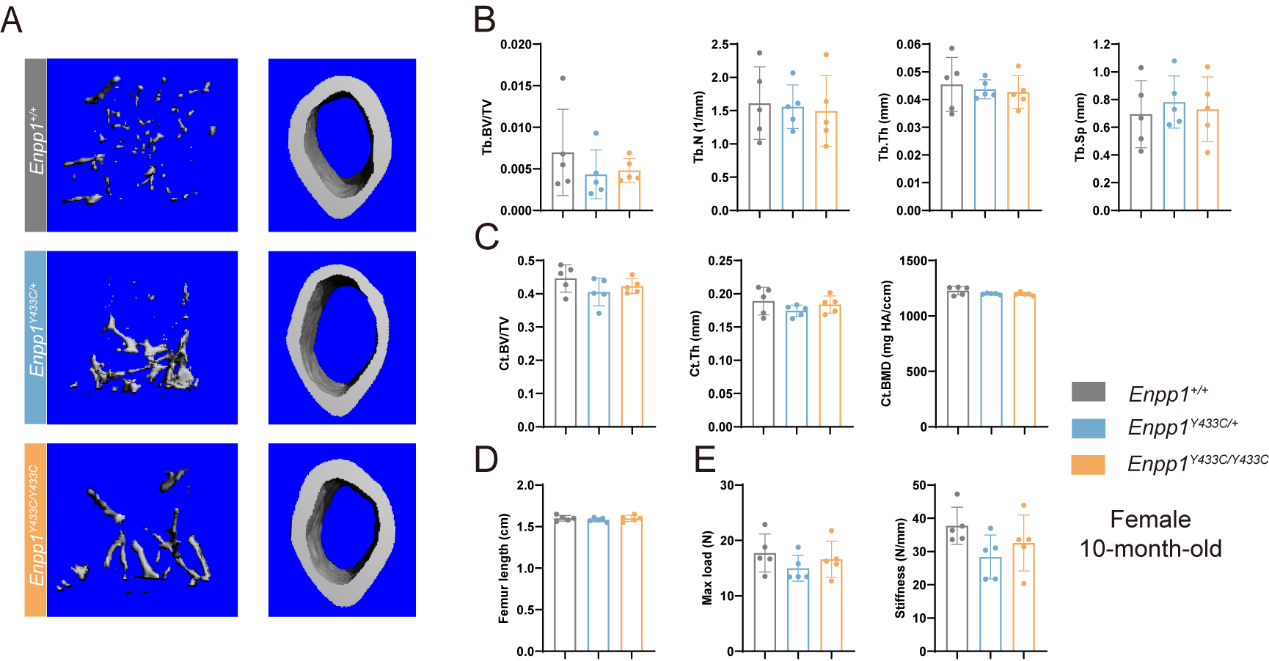
**

**Figure S5. Bone microarchitecture and biomechanics of 10-month-old female mice.**

(A) Three-dimensional reconstructed images of femoral trabecular and cortical bone. (B) Quantification of trabecular BV/TV, trabecular number (Tb.N), trabecular thickness (Tb.Th), Trabecular spacing (Tb.Sp). (C) Quantification of cortical BV/TV, cortical thickness (Ct.Th), and cortical density (Ct. BMD). (D) Femur length. (E) Quantification of femur biomechanical properties (maximum load, stiffness). Data are expressed as mean ± standard deviation(n=5-6). Statistical analysis was performed using one-way ANOVA for comparisons among three groups.
